# Quantitative cross-linking/mass spectrometry using isotope-labelled cross-linkers^☆^

**DOI:** 10.1016/j.jprot.2013.03.005

**Published:** 2013-08-02

**Authors:** Lutz Fischer, Zhuo Angel Chen, Juri Rappsilber

**Affiliations:** a Wellcome Trust Centre for Cell Biology, School of Biological Sciences, University of Edinburgh, Edinburgh EH9 3JR, United Kingdom; b Department of Biotechnology, Technische Universität Berlin, 13353 Berlin, Germany

**Keywords:** Quantitation, Cross-linking, Structural biology, Protein dynamics, Mass spectrometry, Proteomics

## Abstract

Dynamic proteins and multi-protein complexes govern most biological processes. Cross-linking/mass spectrometry (CLMS) is increasingly successful in providing residue-resolution data on static proteinaceous structures. Here we investigate the technical feasibility of recording dynamic processes using isotope-labelling for quantitation. We cross-linked human serum albumin (HSA) with the readily available cross-linker BS3-d0/4 in different heavy/light ratios. We found two limitations. First, isotope labelling reduced the number of identified cross-links. This is in line with similar findings when identifying proteins. Second, standard quantitative proteomics software was not suitable for work with cross-linking. To ameliorate this we wrote a basic open source application, XiQ. Using XiQ we could establish that quantitative CLMS was technically feasible.

**Biological significance:**

Cross-linking/mass spectrometry (CLMS) has become a powerful tool for providing residue-resolution data on static proteinaceous structures. Adding quantitation to CLMS will extend its ability of recording dynamic processes. Here we introduce a cross-linking specific quantitation strategy by using isotope labelled cross-linkers. Using a model system, we demonstrate the principle and feasibility of quantifying cross-linking data and discuss challenges one may encounter while doing so. We then provide a basic open source application, XiQ, to carry out automated quantitation of CLMS data. Our work lays the foundations of studying the molecular details of biological processes at greater ease than this could be done so far.

This article is part of a Special Issue entitled: New Horizons and Applications for Proteomics [EuPA 2012].

## Highlights

• Quantitative cross-linking using isotope labelling is technically feasible
• Commercial cross-linker BS3-d0/4 is suitable for quantitative cross-linking
• XiQ, an open source script to extract quantitative data from raw files
• Cross-link reference data set to test identification and quantitation software
• Isotope labelling reduced the number of identified cross-links

## Graphical abstract

**Figure:**
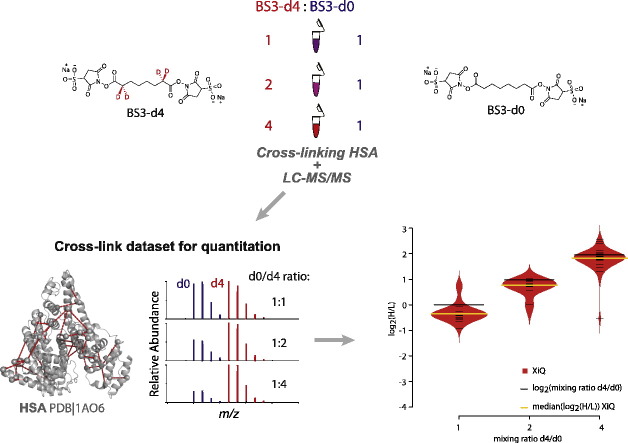

## 1 Introduction

“τά ðντα ìέναι τε πάντα καì μένειν οúδέν” — all entities move and nothing remains still [1]. Dynamic aspects of proteins play a pivotal role in many if not all biological processes. Unfortunately, the analysis of protein dynamics remains a technological challenge, as does the analysis of protein structures. Cross-linking/mass spectrometry (CLMS) is finally emerging after many years of method development as a highly successful tool in the structural analysis of proteins and multi-protein complexes [2,3]. Adding quantitative measurements to CLMS would allow expanding this success to the analysis of protein dynamics, such as conformational changes and protein–protein interaction dynamics.

CLMS currently involves chemical or light-induced cross-linking to covalently fix proximities in proteins or multi-protein complexes [4–6]. Following proteolytic cleavage of the proteins, the cross-linked peptides are identified by mass spectrometry and database searches. An early analysis of a multi-protein complex, the Ndc80 complex (180 kDa, four sub-units) [7], guided crystallisation trials to success [8]. The approach was benchmarked and found to be highly accurate by investigating the 0.5 MDa RNA polymerase II complex (12 sub-units) and provided reciprocal footprints of the transcription factor TFIIF and Pol II [9]. Integration of multiple data sources including cross-linking has also been used to describe phage packaging motor incorporation [10] and has recently led to a model of the proteasome [11], where classical structure determination tools alone have failed for more than a decade. Some of these studies relied on isotope-labelled cross-linkers to enhance the identification success of cross-linked peptides [3,11] while others utilised the high resolution of modern mass spectrometers [7,9].

Adding quantitation to structural analyses by CLMS is an attractive next step that should benefit from the well-established tools of quantitative proteomics. Proteins and their modifications are quantified using isotope labelling [12–15] or label-free approaches. Signal intensities of peptides as measured by mass spectrometry are proportional to peptide concentration and are used routinely for quantitation. Indeed, CLMS has been used to qualitatively reflect conformational changes of single proteins [2,16] or multiple binding sites [9] through observation of conformation-specific cross-links and also in a label free approach for the quantitation of protein–protein interactions [17]. Isotope labelling for quantitation has been explored for cross-link analysis of conformation changes [18]. However, it has yet to be implemented properly for cross-linked peptides. Software tools will likely be of importance, given the key role MaxQuant software [19] has played in making SILAC a success in quantitative proteomics.

Isotope-labelled cross-linkers were introduced some time ago [20,21], and are used extensively to help identify cross-linked peptides, as any doublet resulting from the simultaneous use of light/heavy labelled cross-linker pair indicates a cross-linker-containing peptide [2,7,22–24]. Identification confidence of cross-linked peptides can be increased e.g.: by pre-filtering MS2 spectra for those precursors that were observed as doublets before the search and thus reducing the noise of database searches [7]; by using the mass shifts observed at MS2 level during the search, when both precursors of a doublet were selected for fragmentation [24]; or by adding confidence after the search by checking if identified cross-links were indeed observed as doublets [2]. Unfortunately, using isotope labelled cross-linkers for identification conflicts with their use for quantitation. Selecting both labelled precursors for fragmentation, a need for reliable identification, becomes less likely as ratios deviate from 1:1, which happens when structural changes are being traced. Here, high-resolution mass measurements of cross-linked peptide masses and their fragments may offer a solution. This label-free approach has proven highly successful in CLMS for the purpose of identifying cross-links thus leaving isotope labels for quantitative purposes [9].

The advantage of quantifying by use of cross-linker over other chemical labels, such as isobaric labels (iTRAQ [25] or TMT [26]), is the added identification confidence in knowing, *a priori*, that one looks at a cross-linker containing species. Furthermore, the additional step of adding e.g. iTRAQ or TMT to introduce the label is avoided. Labelled cross-linkers have the same advantage over SILAC [15] as all chemical labelling schemes of not using labelled proteins. Labelling the proteins for cross-linking during synthesis [27] excludes certain biological materials from analysis, such as human serum. Isotope-labelled cross-linkers might therefore be the most general and practical way of introducing isotopes to cross-linked peptides for quantitation. Obviously, this only holds true as long as a given cross-linker is available in isotope-labelled form.

We set out to test isotope-labelled cross-linkers for quantitation. As a model system, we cross-linked human serum albumin and quantified cross-links at different mixing ratios of heavy and light cross-linker. Quantitation was done either manually or by exploiting MaxQuant. As we observed limitations with either method, we developed an application, XiQ, to prove the technical feasibility of the entire approach by combining the accuracy of manual quantitation with the speed of automated quantitation. This also created a reference data set that may be used to test other established quantitation software. The XiQ application and the mass spectrometric raw data used here are available from http://xiq.rappsilberlab.org.

## 2 Methods

### 2.1 Cross-linking and sample preparation

Fifteen microgram aliquots of 0.75 M human serum albumin (HSA) (Sigma) in cross-linking buffer (20 mM HEPES-KOH, 20 mM NaCl, 5 mM MgCl2, pH 7.8) were each cross-linked with mixtures of bis[sulfosuccinimidyl] suberate-d0 (BS3-d0) (Thermo Fisher Scientific) and its deuterated form bis[sulfosuccinimidyl] 2,2,7,7-suberate-d4 (BS3-d4) (Thermo Fisher Scientific). For the purpose of quantitation, BS3-d0 and BS3-d4 were mixed with three molar ratios, 1:1, 1:2 and 1:4. The ratio of BS3-d4:HSA was 4:1 (by mass) in all three mixing ratios. Three replicas were prepared for each ratio. The cross-linking reaction was incubated at room temperature (~ 23 °C) for 1 hour, and quenched by addition of ammonium bicarbonate and incubation for 30 minutes at room temperature. Cross-linked protein samples were isolated on SDS–PAGE gel, and in-gel digested using trypsin following a standard protocol [7]. After digestion, peptide solutions were desalted using self-made C18-StageTips [28], following the published protocol [28] for subsequent mass spectrometric analysis.

### 2.2 Mass spectrometry

We used as analytical column a spray emitter (75-μm inner diameter, 8-μm opening, 250-mm length; New Objectives) that was packed with C18 material (ReproSil-Pur C18-AQ 3 μm; Dr Maisch GmbH, Ammerbuch-Entringen, Germany) by help of an an air pressure pump (Proxeon Biosystems) [29]. Mobile phase A consisted of water and 0.1% formic acid. Mobile phase B consisted of acetonitrile and 0.1% formic acid. Peptides were loaded onto the column with 1% B at 700 nl/min flow rate and eluted at 300 nl/min flow rate with a gradient: 1 minute linear increase from 1% B to 9% B; linear increase to 35% B in 169 minutes; 5 minute increase to 85% B. The eluted peptides were directly sprayed into an LTQ-Orbitrap Velos mass spectrometer (Thermo Fisher Scientific). Mass spectrometric analyses were carried out using a “high-high” acquisition strategy [7,9]. The survey scan (MS) spectra were recorded in the Orbitrap at 100,000 resolution. In each acquisition cycle, the eight most intense signals in the survey scan were isolated with an *m*/*z* window of 2 Th and fragmented with collision-induced dissociation (CID) in the ion trap. 1 + and 2 + ions were excluded from fragmentation. Fragmentation (MS2) spectra were acquired in the Orbitrap at 7500 resolution. Dynamic exclusion was enabled with 90 seconds exclusion time and repeat count equal to 1.

### 2.3 Identification of cross-links

The raw mass spectrometric data files were processed into peak lists using MaxQuant version 1.2.2.5 [19] with default parameters, except “Top MS/MS Peaks per 100 Da” was set to 200. The peak lists were searched against the sequences of HSA using Xi software (ERI, Edinburgh) for identification of cross-linked peptides. Search parameters were as follows: MS accuracy, 6 ppm; MS/MS accuracy, 20 ppm; enzyme, trypsin; specificity, fully tryptic; allowed number of missed cleavages, four; cross-linker, BS3-d0/d4; fixed modifications, carbamidomethylation on cysteine; variable modifications, oxidation on methionine. The linkage specificity for BS3 was assumed to be at lysine, serine, threonine, tyrosine and protein N-termini. Identified candidates of cross-linked peptides were validated by Xi, and only auto-validated cross-linked peptides were used for subsequent quantitation. Distribution of these cross-links was visualised in the crystal structure of HSA (PDB|1AO6) [30] using PyMOL [31]. Distances between alpha-carbons (C-α distances) of cross-linked residues were measured and compared to the maximum cross-linker length, which allowed for further validation of these identified cross-links [9].

### 2.4 Manual quantitation of cross-links

Five cross-linked peptide pairs were quantified manually for each mixing ratio. The cross-links were selected as being amongst those with highest identification confidence in all three replicas. For each cross-linked peptide, the summed intensities of the first three isotope peaks in the isotope cluster of heavy signals and light signals were used to calculate the signal ratio of BS3-d0 cross-linked (light) to BS3-d4 cross-linked (heavy) peptides. We excluded the monoisotopic peak because the relatively small mass difference of 4 Da used here can lead to an overlap of isotope clusters for light and heavy peptides. Peak intensity was defined as the peak area for each isotope peak, and was derived from raw data using Peak detection in Xcalibur (version 2.1.0, Thermo Scientific). Visual inspection ensured that any overlap between light and heavy signals was minimal and that no other signals interfered with the quantitation.

### 2.5 MaxQuant quantitation of cross-links

MaxQuant currently does not support quantitation of cross-linked peptides or indeed of any third-party identifications. Therefore we had to rely on first doing a quantitation that considers doublet signals detected by MaxQuant and then matching these to our results, a process that omits the re-quantitation routine of MaxQuant for identified peptides. We analysed our raw-files using MaxQuant (version 1.2.2.5) allowing for 5 missed cleavages, using a single protein fasta file (HSA), no contaminants, disabled “I = L” and “Filter labelled amino acids,” and multiplicity was set to two. We selected “Lys4” as the heavy label, which corresponds to the mass shift introduced by the labelled cross-linker used in our study. After MaxQuant finished, we concentrated on the allPeptides.txt file. This contains all found “features” in the raw-files and the assigned ratios. These were then mapped back to the identified cross-linked peptides. The ratio for each cross-link site was taken as the median of all supporting ratios for that site.

### 2.6 XiQ quantitation of cross-links

The Xi quantitation application (XiQ) was implemented in C++, following and automating the manual quantitation procedure. It is used in on-going projects in our lab while we are trying to convince the makers of other freely available and more powerful quantitation packages, e.g. MaxQuant [19] and Skyline [32] to make the necessary amendments to their tools that allow cross-link data to be used. XiQ uses the MSFileReader library to access raw-files (Thermo Fisher Scientific). XiQ starts by reading in a tab-delimited file containing the list of identifications (ID list) and opens all provided raw-files. The ID list specifies, for each peptide spectrum match (PSM), the raw-file name, the ms2 scan number, the precursor *m*/*z*, the precursor charge state, the label mass, label count, and quantitation window. For each PSM, XiQ then selects the originating raw-file and in a first step reads out the actual elution time from the scan header of the ms2 event, selected based on the scan number. Additionally, it calculates the expected *m*/*z* value of the mono-isotopic, first, second and third isotope-peak of both signals of the doublet. For the labelled partner the originally defined window is extended in one direction, as deuterium labelling often results in a shift in retention time. If the time window was set originally for the non-labelled peptide it is extended towards earlier elution times and if it was set originally for the deuterium-labelled peptide it is extended to later elution times. The extracted ion current (XIC) is then read out for each isotope peak within the predefined/calculated time window. In each profile it will search for the maximum intensity. From this point XiQ increases the area of the elution peak along the time access until the intensity drops to a value of 10% of the maximum intensity. The peak areas of the first, second and third isotope peak are then summed up for each label-partner and the ratio is defined as heavy area divided by light area. The mono isotopic peak is ignored, as explained above. The calculated ratio is appended to the respective row in the ID list. Site-specific ratios are given by the median of all ratios supporting a given cross-link site. XiQ application is available open source at http://xiq.rappsilberlab.org.

## 3 Results and discussion

### 3.1 HSA analysis

We chose human serum albumin (HSA) as a model system for this study following a number of considerations. It is a medium sized protein (~ 70 kDa) and thus promised to yield richer cross-linking data than smaller proteins, which is desired for statistical analysis. At the same time, HSA does not require additional fractionation steps as larger proteins might. Its crystal structure [30] serves as a reference to support or question the accuracy of any identified cross-links. Furthermore, HSA falls in line with a number of other human serum proteins currently under study in our laboratory and that cannot be obtained as SILAC labelled proteins in their endogenous form. Finally, HSA can be purchased at low cost and in large quantities.

**Fig. 1:**
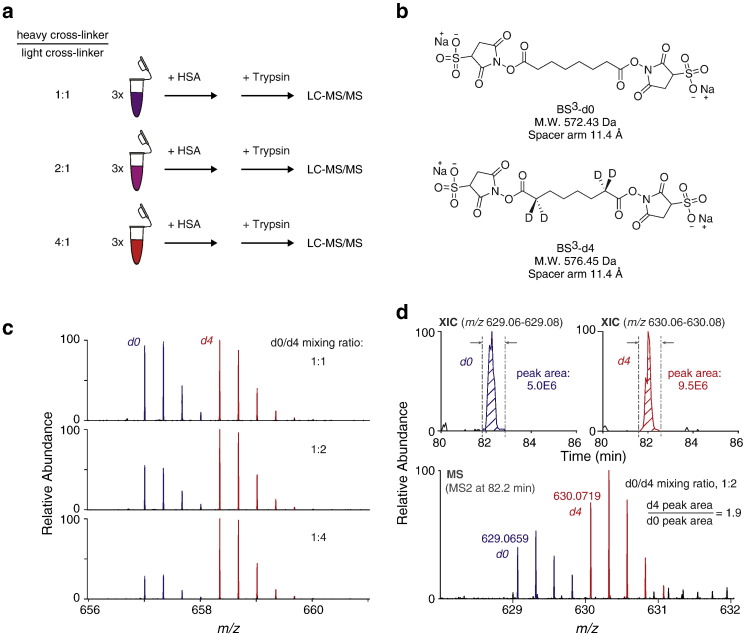
Quantitation of cross-linked peptides using isotope labelled cross-linkers. a. Experiment design. b. Chemical formulae of the cross-linkers BS3-d0 and BS3-d4. c. Doublet signals of a cross-linked peptide, CCK(xl)HPEAK – NLGK(xl)VGSK, detected by mass spectrometry after being cross-linked with mixtures of BS3-d0 and BS3-d4 with molar d0/d4 ratio of 1:1, 1:2, and 1:4. d. d0/d4 ratio for cross-linked peptide DAHK(xl)SEVAHR – FK(xl)DLGEENFK was calculated from the peak area in the extracted ion chromatogram (XIC) for BS3-d0 cross-linked signal divided by the peak area in the XIC of BS3-d4 cross-linked peptide.

Triplicate data sets were generated for three different H/L ratios to serve as model data for quantitation. HSA was cross-linked in solution using 1:1, 1:2 and 1:4 mixtures of bis[sulfosuccinimidyl] suberate (BS3-d0) and its 4 Da heavier deuterated isotopologue BS3-d4 (Fig. 1a–d). Following tryptic digestion and analysis of unfractionated peptides by LC–MS, employing a high-high strategy (i.e. recording MS1 and MS2 by high-resolution mass spectrometry), cross-linked peptides were identified using Xi software. All auto-validated matches were carried forward for quantitative analysis and no attempt was made by manual validation to increase the number of matches. Due to the stringency of auto-validation applied, our results constitute a conservative estimate of the amount of identifiable cross-links in HSA. In triplicate analyses and three mixing ratios, i.e. nine LC–MS runs, we identified 597 spectra of cross-linked peptides using auto-validation in Xi, giving confident evidence to 43 unique cross-links in HSA.

**Fig. 2:**
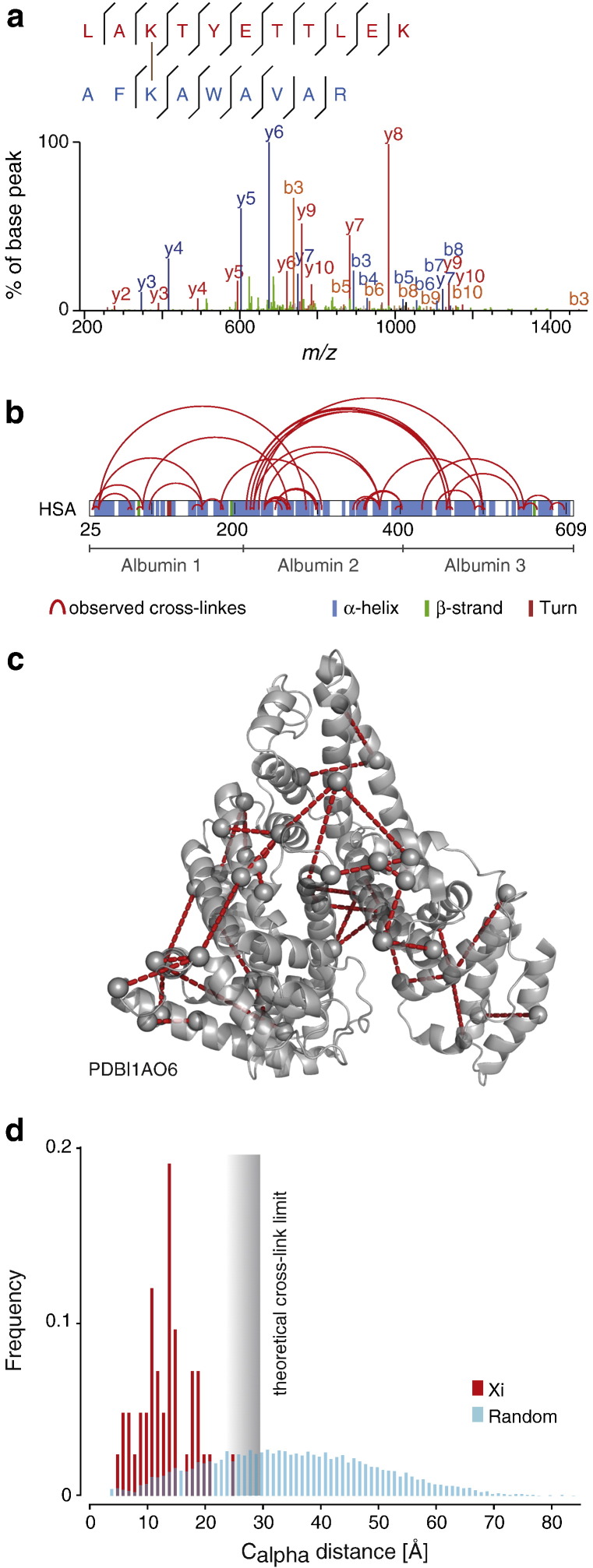
Cross-linking data reflect structure of human serum albumin (HSA). a. Orbitrap fragmentation spectrum of LAK(xl)TYETTLEK – AFK(xl)AWAVAR in xiSPEC (Bukowski-Wills et al., in preparation). b. Cross-link map of HSA using the cross-linker BS3. Cross-links are indicated as curves connecting the linked amino acid positions in HSA. c. Crystal structure of HSA (PDB|1AO6) displayed by PyMOL [31] with cross-links visualised as red dashed lines and alpha-carbons of linked residues as spheres. d. Length distribution of alpha-carbon distances of observed cross-links as measured in the crystal structure of HSA (green) and for random lysine-lysine pairs (grey).

Interestingly, the number of unique cross-links identified depended on the mixing ratio. Using a 1:1 mixture of heavy and light cross-linker resulted in the smallest number of identified cross-links, 17 on average for the triplicate analyses (17, 22, 13). This number increased to 19 cross-links (18, 18, 21) when using 1:2 mixing ratio and to 32 (26, 34, 35) when using a mixing ratio 1:4. This did not affect our quantitative assessment as evaluation was done within each mixing ratio. However, this observation may have implications for cross-link identification strategies. Using H/L versions of cross-linkers to increase identification confidence may come at the expense of missing as much as 50% of the data on cross-links that would otherwise have been observable. Or, in other words, parting from the identification confidence boost of doublets may double the number of observable cross-links. This is in line with similar observations when identifying and quantifying proteins. Xi, used here to identify cross-linked peptides, does not rely on isotope labelling for the identification process but solely on high-resolution data (Fig. 2a).

The observed cross-links distribute over the entire sequence of HSA, connecting all three albumin domains (Fig. 2b). The density of linkages and their distribution suggest the possibility that cross-link data could be used for the assembly of multi-domain protein structures from individual domain structures. In fact, the extensive network links many of the alpha-helices found in HSA (Fig. 2b, c). Modelling software might therefore be assisted in *de-novo* modelling of protein structures [5]. Importantly, none of the 43 cross-links identified by our automated data analysis conflicted with the crystal structure of HSA (Fig. 2d). Considering side chain length of lysines (6–6.5 Å) and the spacer of the cross-linker (11.4 Å) together with some flexibility of the protein in solution results with 25–30 Å as a reasonable cross-link limit, measured between alpha-carbon atoms of lysines. None of the observed cross-links surpass this limit. Furthermore, the cross-link limit is not fulfilled just because of the small size of HSA. A random measurement of alpha-carbon distances between theoretically linkable residues gives clearly a distinct distribution that is in conflict with the cross-link limit. This fully agrees with our prior manual benchmarking of cross-linking data as highly accurate [9].

We concluded that our HSA data set was of high quality, with respect to confidence of identifications, and contained data on sufficient cross-links to statistically assess different routes of extracting quantitative data for cross-links.

### 3.2 Manual peak integration

To test if quantitation was possible at all using our data, and to establish a reference for any automated quantitation algorithm, we manually integrated a number of mass spectrometric signals of cross-linked peptides and calculated their H/L ratio. Five cross-linked peptide pairs were chosen for being amongst those with highest identification confidence in all three mixing ratios and identified in all replicas. Consequently, we had to integrate 15 MS signals for each mixing ratio. This was achieved using Xcalibur to open raw-files and creating an extracted ion chromatogram around the *m*/*z* of the identified cross-link. The peak of the correct elution time, given by the identifying MS2 scan, was then integrated using the peak integration routine of Xcalibur. Peak boundaries were manually determined.

**Fig. 3:**
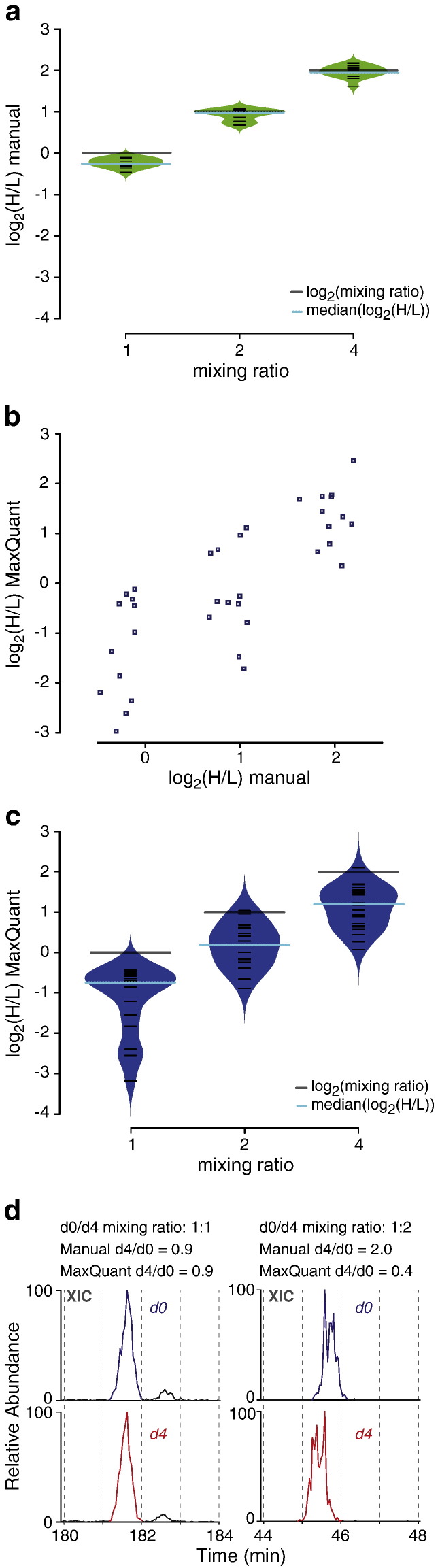
Manual and MaxQuant quantitation of cross-linked peptides. a. Bean plots showing the distribution of manually determined ratios for each mixing ratio. (5 per LC–MS run, three mixing ratios in triplicate). b. H/L ratios for individual doublets of cross-linked peptides, determined by MaxQuant (blue) are plotted against the corresponding manually determined ratios. Only 36 ratios were obtained (see text). c. MaxQuant quantified cross-link sites. Only sites with two or more independent features were considered. The ratio for each site is the median of the ratios of all features supporting the site. d. The impact of the deuterium isotopic effect on MaxQuant based quantitation. In each of the two examples, the XIC of BS3-d0 cross-linked peptide and its BS3-d4 cross-linked counterpart are aligned along the retention time axes. In cases where the deuterium isotope effect causes a large retention time shift between BS3-d0 and BS3-d4 cross-linked peptides (right), the MaxQuant ratio shows a significant difference to manual determined ratio. In contrast, where the deuterium isotope effect is less pronounced, MaxQuant is more in line with manual quantitation. The H/L ratio for a cross-link is the median of all associated evidences. Only cross-links with two or more independent evidences were considered.

Manual quantitation resulted in good agreement between measured and expected ratios (Fig. 3a). All manual values clustered near the experimental mixing ratio, indicating good quantitation accuracy and precision. The median of log2(H/L) was − 0.27, 0.99 and 1.96, respectively, which fell close to the target values of 0, 1 and 2. This demonstrated that neither the small mass difference between BS3-d0 and BS3-d4 nor any elution time shift caused significant interference with the manual quantitation. We noticed, though, that the first isotope of the heavy signal might overlap with the light isotope cluster. For larger peptides, such as cross-linked peptides, the mono-isotopic peak tends to be comparably small and hence the influence of any overlap is potentially significant. We therefore excluded the mono-isotopic peak from our manual quantitation.

### 3.3 Automated quantitation of cross-links

Currently, none of the mainstream quantitation software packages natively support the quantitation of cross-linked peptides. For example, neither Skyline [32] nor MaxQuant [19] accept cross-linked peptides as their input. Nevertheless, we have good experience with using MaxQuant for SILAC based quantitation [33] and also it provides the quantitation of non-identified chromatographic features. We therefore examined to what extend this could be exploited for the quantitation of cross-links. As is the case for SILAC, our approach is based on signal pairs in MS1 that need to be integrated.

MaxQuant performs doublet recognition and peak integration and then outputs the H/L ratios in the “allPeptides.txt” file. There, the ratios are accessible even without using MaxQuant for simultaneous identification, which is not possible in the case of cross-linked peptides. This excludes the re-quantify option of MaxQuant, which is only applicable for peptides identified within the MaxQuant workflow. Directing MaxQuant towards peaks of interest is currently not possible. This means that peaks of cross-links that escaped the doublet detection of MaxQuant are not quantified. Only 19 of 25 identified cross-links in the 1:1 mix ratio data were quantified by MaxQuant. Similar results were obtained for the other mixing ratios, with 22 of 27 (1:2 ratio data) and 28 of 41 (1:4 ratio data) identified cross-links having been quantified by MaxQuant. In some cases, the ratios determined by MaxQuant agreed well with manually determined ratios. However, many MaxQuant ratios disagreed with manual ratios, often being significantly smaller, resulting in overall poor correlation (Pearson correlation 0.79; root-mean-square error, RMSE 1.63). Looking at all quantified cross-links, the ratios were significantly different from the experimental mixing ratios (Fig. 3c). The median of log2(H/L) was − 0.74, 0.15 and 1.19, respectively, which was far from the target values of 0, 1 and 2. The ratios spread over a large range and typically to smaller values. In conclusion, MaxQuant disagreed strongly and inconsistently with our manual quantitation and the expected mixing ratios.

A possible reason for the variable success of MaxQuant could be in the use of a deuterated cross-linker. Deuterium can introduce a large shift in the elution time between labelled and unlabelled cross-linked peptides (Fig. 3d). Future versions of MaxQuant are likely to improve on this (Jürgen Cox, personal communication) and may also include accessing the re-quantify process with peaks selected outside the MaxQuant pipeline. This would be important to ensure determination of ratios for all identified cross-links. For our on-going work on quantitative CLMS, we decided to explore our own software developments.

### 3.4 XiQ quantitation of cross-links

**Supplemental Fig. 1:**
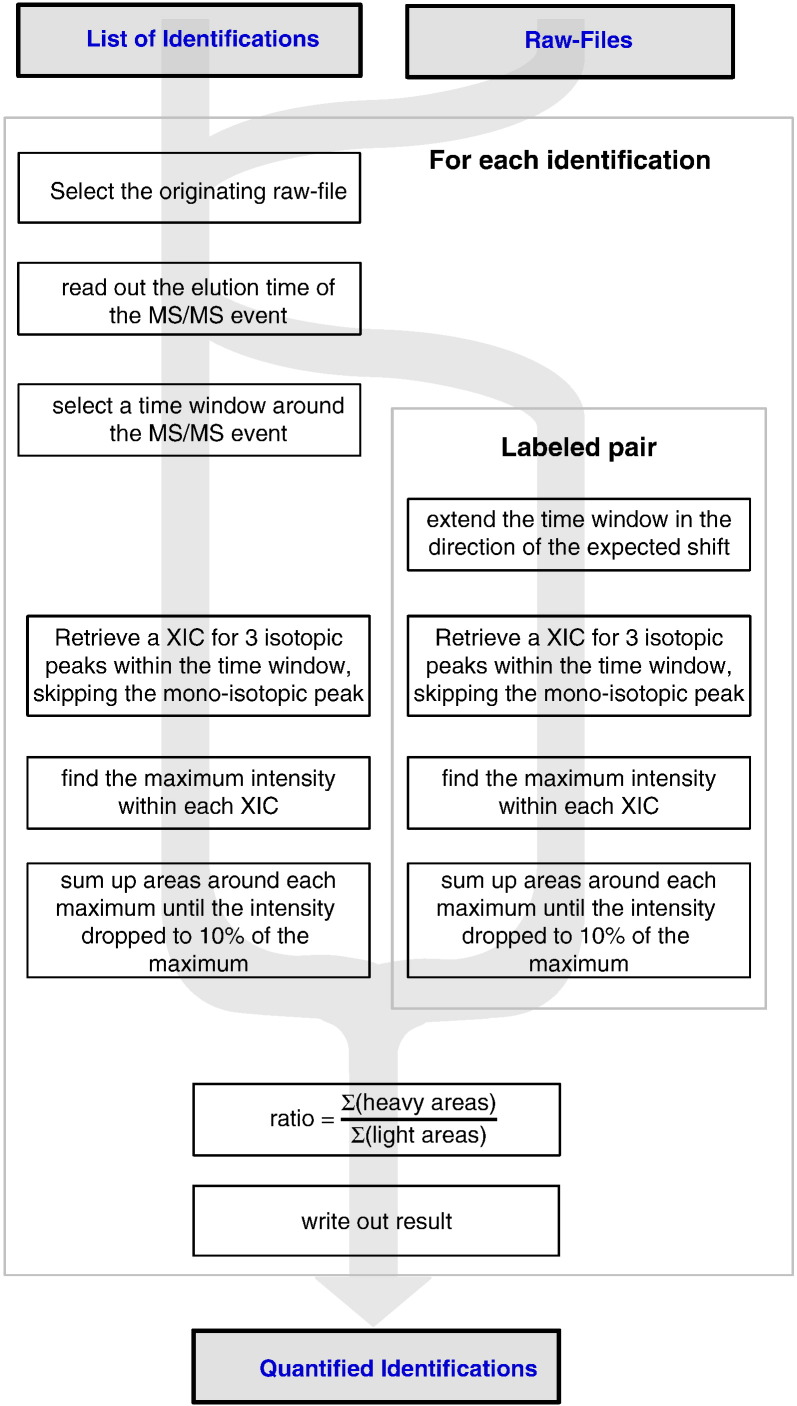
Schematic view of XiQ quantitation.

Given the moderate success of MaxQuant in handling a deuterated cross-linker we decided to develop the open source Xi quantitation software (XiQ). XiQ emulates the steps of the manual quantitation, but without the time involvement and subjective components. The algorithm is described in a flow chart (Fig. S1) and the Methods section. In brief, MSFileReader (Thermo Fisher Scientific) was used to access the mass spectrometric raw-files. The peak volume of the associated precursor signal is extracted for every validated peptide-spectrum match returned from the Xi search algorithm. The same is done for the corresponding doublet signal. Importantly, the elution time window that is considered when looking for the corresponding doublet signal is expanded towards earlier times for the deuterated partner and towards later times for the non-labelled partner. This takes the isotope effect of deuterium on elution time into consideration. Finally, the cross-linked peptide ratio is calculated from the heavy over light signals, considering the first, second and third isotope only, as was done for manual integration.

**Fig. 4:**
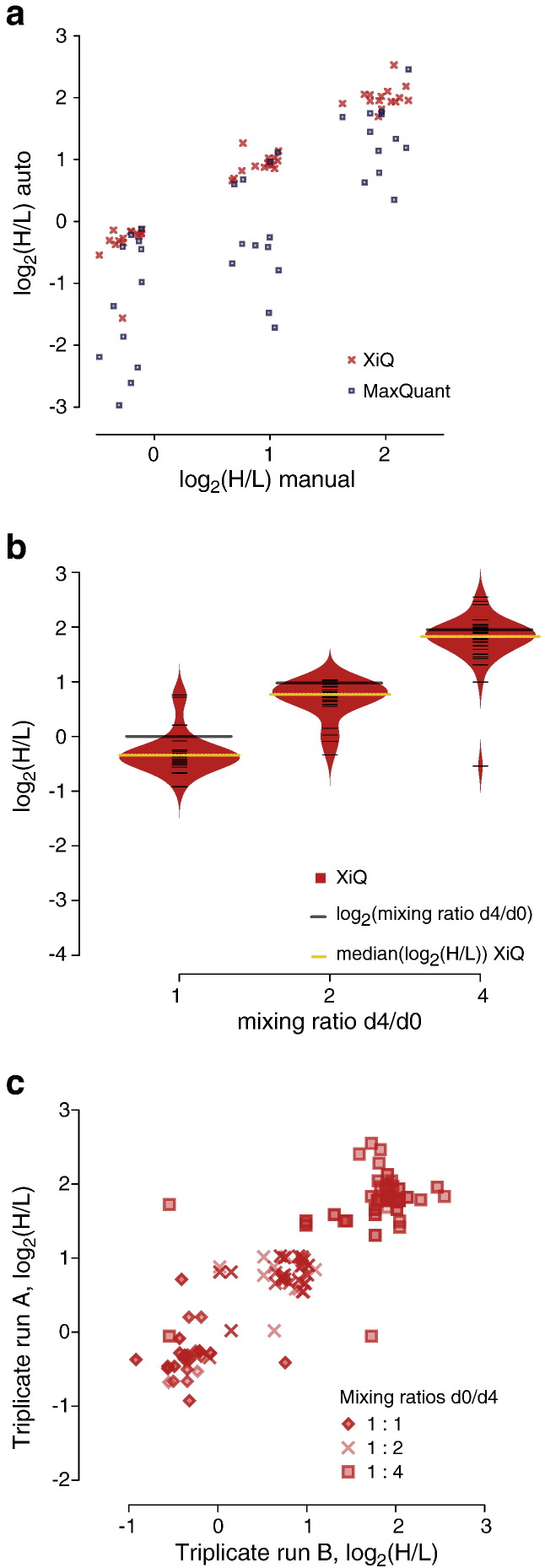
XiQ quantitation of cross-links. a. Comparison of H/L ratios for individual doublets of cross-linked peptides, determined by XiQ (red) and MaxQuant (blue) are plotted versus the corresponding manually determined ratios. For MaxQuant only 36 ratios were obtained (see text) while for XiQ all 45 ratios are displayed (5 per LC–MS run, three mixing ratios in triplicates). b. Bean plots comparing all H/L ratios of cross-links, determined by XiQ (red) with those determined by MaxQuant (blue), for each mixing ratio. Medians are indicated for XiQ (yellow lines) and MaxQuant (blue lines). Black lines mark the experimental mixing ratios. c. XiQ was used for quantitation of all identified cross-links across all mixing ratios and triplicates. For each mixing ratio, all pairwise comparisons of the triplicate analysis are displayed, i.e. LC–MS run 1 versus 2, 1 vs. 3, and 2 vs. 3. A cross-link is not displayed if it was not observed in one run of a comparison pair. The H/L ratio for a cross-link is the median of all associated evidences, at least two.

XiQ compared favourably to manual quantitation. XiQ determined ratios for all 45 manual quantitation points and did so with high agreement to the results of manual quantitation (Fig. 4a). Also, XiQ quantitation achieved fair agreement to the theoretical mixing ratios (Fig. 4b). The medians of log2(H/L) were − 0.36 (− 0.74 MaxQuant), 0.75 (0.15) and 1.81 (1.19), respectively, compared to the target values of 0, 1 and 2. Plotting all triplicate data against each other, i.e. run 1 versus 2, 1 vs. 3 and 2 vs.3, for all mixing ratios, revealed the entire workflow to be repeatable (Fig. 4c), with Pearson correlation 0.89, root-mean-square error (RMSE) 0.22 and coefficient of determination R^2^ 0.77. In summary, XiQ succeeded with delivering reliable and accurate quantitative data for all identified cross-linked peptides.

XiQ does not use any cross-linking specific features. It requires a minimal set of input data, specifically the scan-number of the identifying MS/MS spectrum, precursor *m*/*z* value, charge state, label state (is the *m*/*z* of the heavy or the light precursor), label mass, label count, a time window used to integrate the peaks, and whether a shift in elution time is expected. This makes it possible to use XiQ with any MS1-based labelling scheme that may currently be overlooked by mainstream quantitation software.

## 4 Conclusion

Cross-link data carry valuable information about structure and interactions of proteins. Consequently, quantitative analysis of cross-linked peptides may reveal dynamics of protein conformation and protein interactions. We showed that in principle this could be achieved using stable isotope labels. Unfortunately, mainstream quantitation software currently does not yet handle cross-linked peptides. With XiQ we developed a quantitation application to step in for the time being and to create a full reference data set for testing quantitation software. Furthermore, we provide access to the first mass spectrometric raw data from cross-linking, which allows not only for testing quantitation software but also identification software. This work now lays the foundation and opens the opportunity to venture towards analysing protein dynamics at residue-level resolution by mass spectrometry.

The following are the supplementary data related to this article.

Supplementary material
